# Understanding the pathogenesis of lean non-autoimmune diabetes in an African population with newly diagnosed diabetes

**DOI:** 10.1007/s00125-021-05644-8

**Published:** 2022-02-09

**Authors:** Davis Kibirige, Isaac Sekitoleko, William Lumu, Angus G. Jones, Andrew T. Hattersley, Liam Smeeth, Moffat J. Nyirenda

**Affiliations:** 1 Non-Communicable Diseases Program, Medical Research Council/Uganda Virus Research Institute and London School of Hygiene and Tropical Medicine Uganda Research Unit, Entebbe, Uganda; 2 Department of Non-Communicable Disease Epidemiology, Faculty of Epidemiology and Population Health, London School of Hygiene and Tropical Medicine, London, UK; 3 Department of Medicine, Mengo Hospital, Kampala, Uganda; 4 Institute of Biomedical and Clinical Science, University of Exeter Medical School, Exeter, UK; 5 Department of Diabetes and Endocrinology, Royal Devon and Exeter NHS Foundation Trust, Exeter, UK

**Keywords:** Beta cell dysfunction, Lean non-autoimmune diabetes, Newly diagnosed diabetes sub-Saharan Africa, Type 2 diabetes, Uganda

## Abstract

**Aims/hypothesis:**

Apparent type 2 diabetes is increasingly reported in lean adult individuals in sub-Saharan Africa. However, studies undertaking robust clinical and metabolic characterisation of lean individuals with new-onset type 2 diabetes are limited in this population. This cross-sectional study aimed to perform a detailed clinical and metabolic characterisation of newly diagnosed adult patients with diabetes in Uganda, in order to compare features between lean and non-lean individuals.

**Methods:**

Socio-demographic, clinical, biophysical and metabolic (including oral glucose tolerance test) data were collected on 568 adult patients with newly diagnosed diabetes. Participants were screened for islet autoantibodies to exclude those with autoimmune diabetes. The remaining participants (with type 2 diabetes) were then classified as lean (BMI <25 kg/m^2^) or non-lean (BMI ≥25 kg/m^2^), and their socio-demographic, clinical, biophysical and metabolic characteristics were compared.

**Results:**

Thirty-four participants (6.4%) were excluded from analyses because they were positive for pancreatic autoantibodies, and a further 34 participants because they had incomplete data. For the remaining 500 participants, the median (IQR) age, BMI and HbA_1c_ were 48 years (39–58), 27.5 kg/m^2^ (23.6–31.4) and 90 mmol/mol (61–113) (10.3% [7.7–12.5]), respectively, with a female predominance (approximately 57%). Of the 500 participants, 160 (32%) and 340 (68%) were lean and non-lean, respectively. Compared with non-lean participants, lean participants were mainly male (60.6% vs 35.3%, *p*<0.001) and had lower visceral adiposity level (5 [4–7] vs 11 [9–13], *p*<0.001) and features of the metabolic syndrome (uric acid, 246.5 [205.0–290.6] vs 289 [234–347] μmol/l, *p*<0.001; leptin, 660.9 [174.5–1993.1] vs 3988.0 [1336.0–6595.0] pg/ml, *p*<0.001). In addition, they displayed markedly reduced markers of beta cell function (oral insulinogenic index 0.8 [0.3–2.5] vs 1.6 [0.6–4.6] pmol/mmol; 120 min serum C-peptide 0.70 [0.33–1.36] vs 1.02 [0.60–1.66] nmol/l, *p*<0.001).

**Conclusions/interpretation:**

Approximately one-third of participants with incident adult-onset non-autoimmune diabetes had BMI <25 kg/m^2^. Diabetes in these lean individuals was more common in men, and predominantly associated with reduced pancreatic secretory function rather than insulin resistance. The underlying pathological mechanisms are unclear, but this is likely to have important management implications.

**Graphical abstract:**

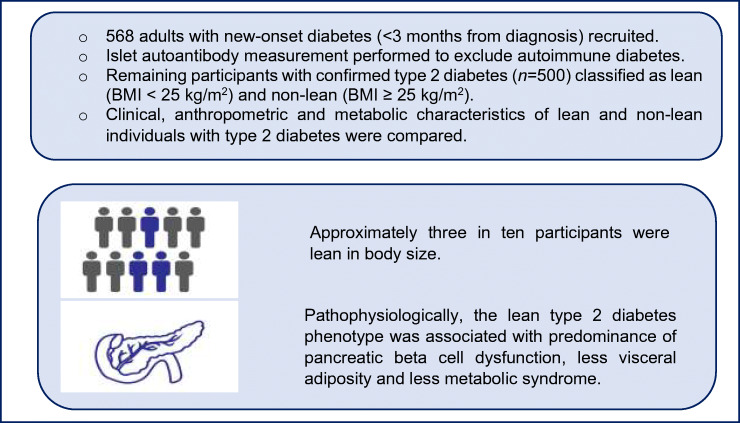

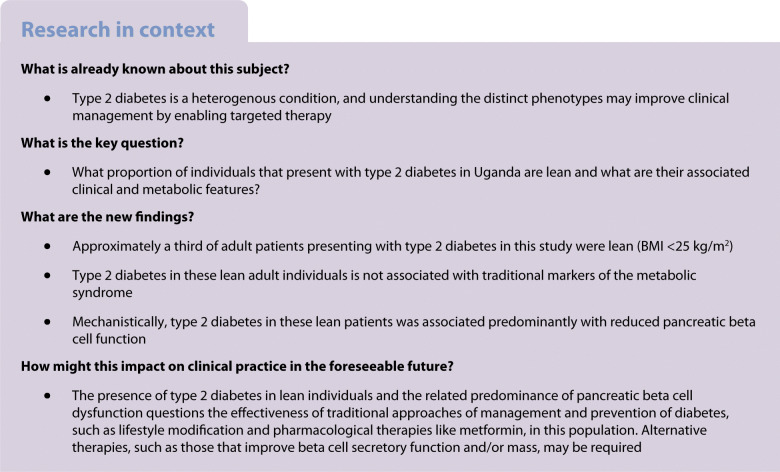



## Introduction

Type 2 diabetes has reached epidemic proportions globally. In sub-Saharan Africa, it remains a major and rapidly growing problem, posing a significant public health challenge and causing a considerable strain on the healthcare systems [1]. Overweight, obesity, rapid urbanisation and changes in lifestyle are implicated in the increasing burden of type 2 diabetes globally [1, 2]. Despite obesity and overweight being well-documented risk factors of type 2 diabetes, there is accumulating evidence, particularly from low- and middle-income countries, that type 2 diabetes can also develop in lean individuals [3–10].

Most of the evidence on type 2 diabetes in lean individuals has originated from South Asian populations, where diabetes is commonly seen in people with normal BMI. Initial studies showed that, although BMI was normal, these individuals typically had increased waist circumferences, WHR, total body and visceral adiposity, and ectopic fat deposition, with a metabolic profile characterised by an atherogenic lipid profile (low HDL-cholesterol, high VLDL-cholesterol and triacylglycerol concentrations) [11–15]. This was suggestive of insulin resistance as the underlying pathogenic mechanism. However, recent evidence has shown that beta cell secretory dysfunction may be the primary pathogenic defect in such individuals [6, 7, 11, 12, 15–19].

In contrast, studies undertaking robust clinical and metabolic characterisation of true new-onset type 2 diabetes (where islet cell autoimmunity has been excluded) in lean individuals in sub-Saharan Africa are few in number. To add to the existing literature, we conducted the Uganda DIabetes Phenotype (UDIP) study to examine the socio-demographic, clinical, biophysical and metabolic characteristics of adult patients with newly diagnosed type 2 diabetes in Uganda, in order to compare the features associated with diabetes in lean and non-lean individuals.

## Methods

### Study setting and participants

This study was conducted in seven tertiary public and private not-for-profit mission or church-founded hospitals in Central and Southwestern Uganda serving urban, peri-urban and rural populations between February 2019 and October 2020. About 85% of the Ugandan population receives medical care from public and private not-for-profit hospitals.

We consecutively recruited adult patients aged ≥18 years with a recent diagnosis of diabetes. The diagnosis of diabetes had been made by clinicians at various general outpatient clinics, based on results for fasting blood glucose, random blood glucose and HbA_1c_ measurement. After diagnosis, patients are referred to the diabetes clinics for further management.

Patients were recruited within 3 months of diagnosis when they attended the outpatient diabetes clinics for routine clinical reviews. Critically ill patients who required urgent hospitalisation for medical treatment were not immediately recruited into the study but were invited to enrol at least 2 weeks after discharge from hospital (but within 3 months of diagnosis) when they re-attended the diabetes clinics in a stable condition. Both treatment-naive patients and those who had commenced glucose-lowering therapy were allowed to participate in the study. Pregnant women were excluded. All recruited study participants were black Africans of Ugandan origin. A total of 568 adult patients with newly diagnosed diabetes were recruited into the study.

### Assessment of socio-demographic, clinical and biophysical characteristics

All study participants were assessed after an overnight fast of ≥8 h. Relevant socio-demographic variables (age at diagnosis, sex, residence, level of education, family history of diabetes, smoking and alcohol intake status) and clinical data (history of admission at diagnosis, presence of urine or serum ketones at admission, use of diabetes and ancillary drugs, coexisting medical comorbidities) were collected by the research team using a pre-tested case report form. This was followed by biophysical measurements including resting blood pressure and relevant anthropometric measurements (weight, height, waist circumference and hip circumference for calculation of BMI, WHR and waist circumference:height ratio [WHtR]) according to standardised study procedures. Body composition (total body fat and visceral fat levels) was evaluated by bioimpedance analysis using an OMRON BF511 body composition monitor (Omron Healthcare, Tokyo, Japan). Hypertension was defined as systolic BP ≥140 mmHg and/or diastolic BP ≥90 mmHg on clinical examination or a self-reported history of pre-existing hypertension either on antihypertensive therapy or without treatment [2].

### Assessment of metabolic characteristics

A fasting blood sample was collected for measurement of fasting blood glucose, HbA_1c_, insulin, C-peptide and lipid profile, uric acid, leptin, and three pancreatic autoantibodies (GAD autoantibodies [GADA], zinc transporter eight autoantibodies [ZnT8-A] and autoantibody to the protein tyrosine phosphatase [IA-2A]). This was followed by a 75 g OGTT, with blood samples drawn again 30 and 120 min after glucose ingestion to determine the serum glucose, insulin and C-peptide concentrations at those two time points.

### Laboratory measurements and assessment of markers of pancreatic beta cell function, insulin resistance and sensitivity

All the laboratory tests were performed at the ISO-certified clinical chemistry laboratory at Medical Research Council/Uganda Virus Research Institute and London School of Hygiene and Tropical Medicine Uganda Research Unit, Entebbe, Uganda, using electro-chemiluminescence immunoassays manufactured by Roche (Germany) using a Cobas 6000 C-model SN 14H3–15 machine (Hitachi High Technologies, Japan). Pancreatic autoantibody testing was performed using autoantibody ELISA kits from RSR (UK) on the Dynex DS2 ELISA robot (Dynex Technologies, UK).

The HOMA2 calculator (Diabetes Trial Unit, University of Oxford, UK) was used to calculate the insulin resistance (HOMA2-IR) and the pancreatic beta cell function (HOMA2-%B) [20]. Insulinogenic index (IGI) as an optimum marker of pancreatic beta cell function was calculated using this formula: IGI = (30 min insulin − 0 min insulin in pmol/l)/(30 min glucose – 0 min glucose in mmol/l) [21]. The quantitative insulin sensitivity check index (QUICKI) to indicate insulin sensitivity was calculated from fasting serum glucose and insulin concentrations using the online QUICKI calculator [22].

### Exclusion of patients with pancreatic autoimmunity

Pancreatic autoantibody testing was performed in all participants to exclude those with islet autoantibody positivity as a marker of pancreatic autoimmunity. Pancreatic autoantibody positivity was defined as levels of GADA >34 U/ml or IA-2A > 58 U/ml or ZnT8-A > 67.7 U/ml. These thresholds represent the 97.5th percentile for 600 randomly selected healthy Ugandan adults without diabetes enrolled in the Medical Research Council/Uganda Virus Research Institute and London School of Hygiene and Tropical Medicine Uganda Research Unit general population cohort (Balungi et al, unpublished data).

After excluding those with pancreatic autoimmunity, the remaining participants with type 2 diabetes were classified as lean and non-lean based on the traditional BMI cut-offs of <25 and ≥25 kg/m^2^, respectively, because there are no Africa- or Uganda-specific BMI cut-offs to define obesity. The socio-demographic, clinical, biophysical and metabolic characteristics of both groups were then compared.

### Ethical approval

The study received ethical approval from the research ethics committee of the Uganda Virus Research Institute (GC/127/18/05/650) and Uganda National Council of Science and Technology (HS 2431). All participating study sites offered administrative approval prior to initiation of the study. All study participants recruited into the study provided written informed consent.

### Statistical analysis

The categorical and continuous variables describing all the study participants are expressed as percentages and medians with inter-quartile range (IQR), respectively. The differences in the socio-demographic, clinical and metabolic characteristics between the lean and non-lean participants were analysed using the χ^2^ test for categorical data and the Kruskal–Wallis test for continuous data. Because of comparison of multiple variables between the lean and non-lean participants, the Bonferroni correction (adjusted *p* value = set significance α/number of variables tested) was used [23]. With the 47 variables to be compared between the two groups and the set significance α of 0.05, the adjusted *p* value to signify statistical significance became 0.001. All analyses were performed using STATA statistical software version 15 (StataCorp, USA). A *p* value <0.05 was considered statistically significant.

## Results

A total of 568 adult patients with newly diagnosed diabetes were recruited. Complete data on islet autoantibody status was available in 534 patients (94%) with newly diagnosed diabetes. Of these, 34 participants (6.4%) were excluded because they were positive for at least one of the three pancreatic autoantibodies. Applying the BMI cut-offs of <25 and ≥25 kg/m^2^ in the remaining 500 participants, 160 (32%, 95% CI 27.9–36.3%) and 340 (68%, 95% CI 63.7–72.1%) were lean and non-lean, respectively.

The socio-demographic, clinical, biophysical and metabolic characteristics of all study participants with newly diagnosed diabetes are summarised in Table 1.

**Table 1:** Socio-demographic, clinical, anthropometric and metabolic characteristics of all study participants, and for lean and non-lean participants separately

Characteristic	All study participants (*n* = 500)	Lean participants (*n*=160)	Non-lean participants (*n*=340)	*p* value (lean vs non-lean)
Socio-demographic and clinical data				
Age (years)	48 (39–58)	48 (37–58)	48 (40–57)	1.00
Sex				
Male	217 (43.4)	97 (60.6)	120 (35.3)	<0.001***
Female	283 (56.6)	63 (39.4)	220 (64.7)	
Residence^a^				
Urban	370 (74.1)	119 (74.8)	251 (73.8)	0.77
Rural	127 (25.5)	39 (24.5)	88 (25.9)	
Prior admission at diagnosis^b^	202 (40.6)	81 (50.6)	121 (35.8)	0.003
Presence of urine or serum ketones at admission^c^	70 (30.7)	34 (39.1)	36 (25.5)	0.02
Treatment used^d^				
Diet	18 (3.6)	3 (1.9)	15 (4.4)	0.16
Metformin	401 (80.2)	114 (71.3)	287 (84.4)	0.001***
Sulfonylureas	191 (38.2)	49 (30.6)	142 (41.8)	0.02
Insulin	134 (26.8)	68 (42.5)	66 (19.4)	<0.001***
Self-reported HT comorbidity^e^	171 (35.0)	39 (24.8)	132 (39.9)	0.002
*Acanthosis nigricans* present	89 (17.8)	17 (10.6)	72 (21.2)	0.004
Systolic BP (mmHg)	126 (115–137)	123 (109–133)	127 (117–139)	0.08
Diastolic BP (mmHg)	84 (77–91)	80 (74–87)	85 (79–93)	<0.001***
HT on clinical examination (SBP ≥ 140 and/or DBP ≥ 90 mmHg)	178 (35.6)	43 (27.0)	135 (39.8)	0.006
Anthropometric data				
Weight (kg)	72.0 (62.5–82.0)	58.2 (52.2–65.0)	77.2 (71.0–86.6)	<0.001***
Height (cm)	162.0 (156.4–167.2)	163.1 (158.0–168.6)	161.1 (156.0–166.5)	0.06
BMI (kg/m^2^)	27.5 (23.6–31.4)	22.2 (20.3–23.5)	30.1 (27.3–33.1)	<0.001***
WC (cm)	96.0 (87.0–104.8)	83.0 (77.0–90.0)	101.6 (95.0–108.0)	<0.001***
HC (cm)	103.0 (96.0–111.5)	93.5 (88.0–98.0)	107.0 (102.0–116.0)	<0.001***
WHR	0.92 (0.88–0.96)	0.90 (0.85–0.95)	0.93 (0.89–0.97)	<0.001***
WHtR	0.59 (0.53–0.65)	0.51 (0.48–0.55)	0.63 (0.58–0.68)	<0.001***
Total body fat (%)	36.4 (26.5–45.3)	22.5 (16.1–31.6)	42.0 (32.8–47.9)	<0.001***
Visceral fat level	9 (7–12)	5 (4–7)	11 (9–13)	<0.001***
Metabolic data				
TC (mmol/l)	4.0 (3.3–5.0)	3.8 (3.1–4.7)	4.2 (3.4–5.0)	0.03
HDLC (mmol/l)	1.0 (0.7–1.2)	1.0 (0.7–1.2)	0.9 (0.8–1.2)	0.21
TGL (mmol/l)	1.3 (1.0–1.8)	1.2 (0.9–1.7)	1.4 (1.1–1.9)	0.02
LDLC (mmol/l)	2.6 (1.9–3.4)	2.4 (1.7–3.3)	2.6 (2.0–3.5)	0.11
Non-HDLC (mmol/l)	3.0 (2.4–3.8)	2.8 (2.2–3.6)	3.1 (2.5–3.9)	0.03
TC/HDLC	4.2 (3.4–5.3)	3.9 (3.3–5.0)	4.4 (3.5–5.4)	0.002
TGL/HDLC	1.4 (1.0–2.2)	1.3 (0.9–2.0)	1.5 (1.0–2.3)	0.05
Uric acid (μmol/l)	273.0 (222.0–335.0)	246.5 (205.0–290.6)	289.0 (234.0–347.0)	<0.001***
Leptin (pg/ml)	2538.3 (606.8–5477.0)	660.9 (174.5–1993.1)	3988.0 (1336.0–6595.0)	<0.001***
HbA_1c_ (mmol/mol)	90 (61–113)	99 (58–121)	86 (61–110)	0.02
HbA_1c_ (%)	10.3 (7.7–12.5)	11.1 (7.4–13.2)	10.0 (7.7–12.2)	0.02
Fasting blood glucose (mmol/l)	8.6 (6.2–13.4)	9.1 (5.8–14.5)	8.4 (6.2–12.8)	0.28
Fasting serum insulin (pmol/l)	40.97 (20.83–73.61)	29.17 (14.58–44.44)	48.61 (25.69–85.42)	<0.001***
Fasting serum C-peptide (nmol/l)	0.46 (0.27–0.70)	0.33 (0.20–0.53)	0.53 (0.36–0.76)	<0.001***
30 min blood glucose (mmol/l)	13.0 (10.0–18.3)	13.5 (9.9–20.0)	12.6 (10.0–17.5)	0.19
30 min serum insulin (pmol/l)	77.08 (38.19–156.25)	52.08 (21.53–100.00)	95.83 (45.83–178.47)	<0.001***
30 min C-peptide (nmol/l)	0.70 (0.36–1.09)	0.50 (0.23–0.83)	0.80 (0.50–1.23)	<0.001***
120 min blood glucose (mmol/l)	17.2 (12.3–23.3)	18.8 (14.0–25.2)	16.5 (11.8–22.0)	0.02
120 min serum insulin (pmol/l)	95.14 (47.92–188.19)	61.81 (29.86–123.61)	115.28 (56.25–227.08)	<0.001***
120 min serum C-peptide (nmol/l)	0.93 (0.50–1.59)	0.70 (0.33–1.36)	1.02 (0.60–1.66)	<0.001***
HOMA2-IR	1.21 (0.77–2.03)	0.89 (0.65–1.58)	1.32 (0.84–2.17)	0.001***
QUICKI	0.35 (0.31–0.42)	0.37 (0.33–0.50)	0.34 (0.31–0.39)	0.001***
HOMA2-%B	43.1 (20.7–77.6)	33.3 (15.5–75.8)	44.3 (23.4–77.7)	0.11
Oral IGI (pmol/mmol)	1.3 (0.5–3.9)	0.8 (0.3–2.5)	1.6 (0.6–4.6)	0.001***

Data are presented in form of percentages for categorical variables and as median with IQR for continuous variables

^a^ One participant (in the lean category) had missing data on residence

^b^ Two participants (in the non-lean category) had missing data on prior admission status

^c^ Percentages are calculated using the numbers of people with prior history of admission at diagnosis as the denominator; differences are due to missing data

^d^ Used as monotherapy or in combination

^e^ Three participants in the lean category and nine participants in the non-lean category had missing data on self-reported HT comorbidity

DBP, Diastolic blood pressure; HC, hip circumference; HDLC, HDL-cholesterol; HT, hypertension; LDLC, LDL-cholesterol; SBP, systolic blood pressure; TC, total cholesterol; TGL, triacylglycerol; WC, waist circumference

***p<0.001

### Socio-demographic, clinical and biophysical characterisation of the lean and non-lean participants

The socio-demographic, clinical, biophysical and metabolic characteristics of the lean and non-lean participants are summarised in Table 1.

Compared with those who were non-lean, lean participants were predominantly male (60.6% vs 35.3%, *p*<0.001) and were started on insulin therapy at diagnosis (42.5% vs 19.4%, *p*<0.001). No difference in age at diagnosis was noted between the two subgroups (48 [37–58] vs 48 [40–57] years, *p*=1.00).

Lean participants also had significantly lower median (IQR) waist circumference (83.0 [77.0–90.0] cm vs 101.6 [95.0–108.0] cm, *p*<0.001), WHR (0.90 [0.85–0.95] vs 0.93 [0.89–0.97], *p*<0.001), WHtR (0.51 [0.48–0.55] vs 0.63 [0.58–0.68], *p*<0.001), total body fat (22.5 [16.1–31.6] % vs 42.0 [32.8–47.9] %, *p*<0.001) and visceral fat level (5 [4–7] vs 11 [9–13], *p*<0.001).

### Metabolic characterisation of lean and non-lean participants

**Fig. 1:**
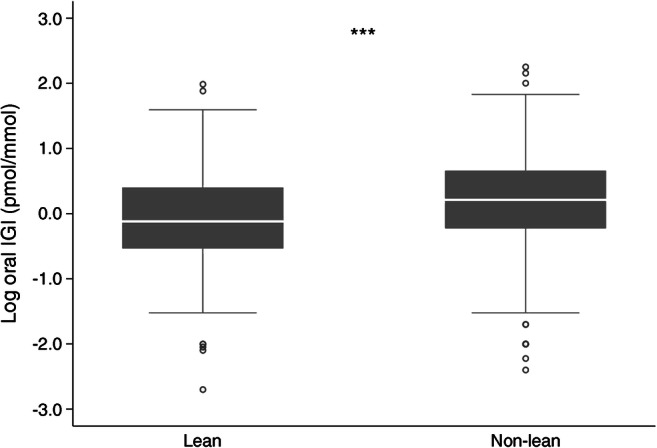
Comparison of log oral IGI among the lean and non-lean participants. ***p<0.001

**Fig. 2:**
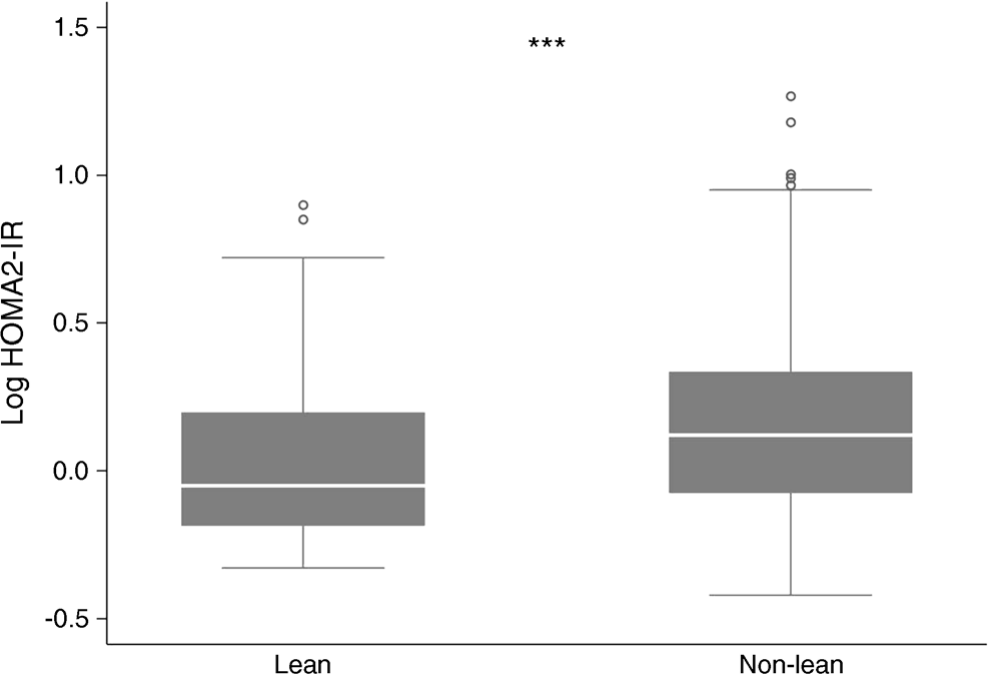
Comparison of log HOMA-IR between the lean and non-lean participants. ***p<0.001

Biochemically, compared with non-lean participants, lean participants had significantly lower median fasting insulin (29.17 [14.58–44.44] pmol/l vs 48.61 [25.69–85.42] pmol/l, *p*<0.001), fasting C-peptide (0.33 [0.20–0.53] nmol/l vs 0.53 [0.36–0.76] nmol/l, *p*<0.001), 30 min insulin (52.08 [21.53–100.00] pmol/l vs 95.83 [45.83–178.47] pmol/l, *p*<0.001), oral IGI (0.8 [0.3–2.5] pmol/mmol vs 1.6 [0.6–4.6] pmol/mmol, *p*=0.001) and HOMA2-IR (0.89 [0.65–1.58] vs 1.32 [0.84–2.17], *p*=0.001). The oral IGI, as an optimum marker of beta cell function, and HOMA2-IR of the lean and non-lean participants are summarised as box plots in Figs 1 and 2, respectively.

Lean participants were also more likely to have lower circulating concentrations of biomarkers of the metabolic syndrome such as leptin (660.9 [174.5–1993.1] pg/ml vs 3988.0 [1336.0–6595.0] pg/ml, *p*<0.001), and uric acid (246.5 [205.0–290.6] μmol/l vs 289.0 [234.0–347.0] μmol/l, *p*<0.001).

## Discussion

In this study of well-characterised adult patients with newly diagnosed type 2 diabetes, being lean in body size (BMI <25 kg/m^2^) was relatively common, occurring in approximately a third of participants. These individuals showed biochemical features that are consistent with pancreatic beta cell dysfunction rather than insulin resistance. These findings are in direct contrast with the conventional picture of diabetes in adulthood, consisting of overweight or obesity and insulin resistance, with pancreatic beta cell failure occurring later during the condition.

However, our study findings are in accordance with other data emerging from sub-Saharan Africa that show a relatively high proportion of those with type 2 diabetes are thin. [5, 24–26]. For example, three large population-based studies performed in Uganda [24, 25] and Ethiopia [26] reported that type 2 diabetes in lean individuals accounted for approximately 60% of all cases of type 2 diabetes. These are significantly higher rates than observed in our study, perhaps because more that 70% of the total participants in these surveys had a BMI <25 kg/m^2^. The Research on Obesity and Diabetes among African Migrants (RODAM) study, which was performed in native and migrant Ghanaians, also reported high proportions of type 2 diabetes (55.4% and 35.6% in rural and urban native Ghanaians, respectively) in individuals with BMI <25 kg/m^2^.

In contrast, a lower frequency of type 2 diabetes in individuals with BMI <25 kg/m^2^ has been reported in large-scale studies in people of European extraction or Asian and Hispanic populations, where prevalence rates ranged between 5 and 23.5% [4, 6, 8–10]. More importantly, our study adds to the increasing evidence that type 2 diabetes is a heterogenous disorder whose pathogenesis differs across populations or ethnicities [27].

South Asians have also been shown to develop type 2 diabetes at lower BMI values than people of European extraction, which has led to use of Asia-specific lower BMI thresholds for defining obesity [28]. Despite low BMI, South Asians have been shown to have a propensity towards central obesity, increased markers of the metabolic syndrome (ectopic fat, atherogenic lipid profile and higher systolic and diastolic pressures) and lower circulating levels of adiponectin [11, 12, 14, 15]. In contrast, anthropometric (waist circumference, WHR, WHtR) and body composition measurements (total body fat and visceral fat levels) in the lean participants in our study did not indicate increased adiposity. In addition, participants in our study had significantly lower prevalence of other features of the metabolic syndrome (such as hyperleptinaemia and hyperuricaemia) and low HOMA2-IR compared with the non-lean participants. These findings suggest that insulin resistance is not the major underlying mechanism for type 2 diabetes in lean patients in Uganda. Instead, pancreatic beta cell secretory dysfunction, as reflected by a lower oral IGI, fasting and 120 min C-peptide levels and greater blunting of both the first and delayed phases of insulin secretion appears to be the predominant primary pathophysiological defect.

Although the presence of central obesity and related features in the South Asian population with lean type 2 diabetes led to the speculation that insulin resistance is the driving pathogenic mechanism, more recent studies have established that pancreatic beta cell dysfunction is the primary defect [6, 7, 15, 18, 19]. In this respect, the lean type 2 diabetes phenotype in South Asia may share a common aetiological mechanism with our Ugandan adult population. Differences in manifestation of diabetes may be influenced by other local factors, such as dietary patterns. Phenotypic features similar to those of South Asians, such as increased abdominal visceral fat content relative to abdominal subcutaneous fat, low BMI at diagnosis and pancreatic beta cell dysfunction as the predominant pathogenic defect, have also been described in East Asians with type 2 diabetes [29–31].

The mechanisms that lead to pancreatic beta cell failure in lean patients with type 2 diabetes are not clear. One hypothesis, the Developmental Origins of Health and Disease (DOHaD) concept, suggests that undernutrition during critical windows of development may impair organ development, resulting in a naturally existing small beta cell mass, or may impair beta cell replication or neogenesis or induce metabolic/epigenetic changes that ultimately lead to increased risk of metabolic diseases such as type 2 diabetes later in life [32, 33]. Undernutrition, particularly during pregnancy and in early childhood, remains common in sub-Saharan Africa, and yet coexists with the global obesity epidemic. The combination of adverse early-life influences and a demographic shift towards urbanisation and westernisation may fuel the increased susceptibility to type 2 diabetes in sub-Saharan Africa.

Sub-Saharan Africa is also rich in genetic heterogeneity, which may influence the pathogenesis and clinical course of diabetes in African populations. For example, a recent genome-wide association study of 5231 African patients with type 2 diabetes identified a novel significant locus for type 2 diabetes called *ZRANB3* (encoding zinc finger RANBP2-type containing 3). This gene product, through apoptotic events, leads to reduced pancreatic beta cell mass [34]. The gene encoding transcription factor 7-like 2 (*TCF7L2*), which is known to affect pancreatic secretory function, has also been described in African populations with type 2 diabetes [35].

The relatively high prevalence of type 2 diabetes in lean individuals in sub-Saharan Africa has major implications for screening or diagnosis because age and BMI are widely used to clinically differentiate between type 1 and type 2 diabetes. Each type has a different management approach (one requiring immediate insulin treatment to save life and the other easily managed by lifestyle and/or oral hypoglycaemic therapy). Similarly, age and BMI thresholds are used to decide whom to screen for type 2 diabetes as well as to guide prevention and treatment strategies. The diabetes management approach that involves use of an algorithm that begins with lifestyle intervention or metformin as the first-line agent are based on evidence of benefit in studies of mainly obese or overweight patients of European extraction with predominant insulin resistance in high-income countries [36–38]. It is unclear whether these therapeutic interventions have the same effectiveness in sub-Saharan Africa where many adult patients are relatively young, lean in body size and not insulin-resistant.

Our study had a number of strengths. It had a large sample size and was undertaken across multiple tertiary hospitals recruiting only newly diagnosed patients (within three months of diagnosis). This minimised the potential confounding effects of long-term complications of the disease. We used rigorous study protocols, and screened participants for the presence of three common islet autoantibodies (using local population-derived diagnostic cut-off points for positivity) to exclude those with presumed autoimmune diabetes. We also used one of the highest performing pancreatic autoantibody assays as assessed by the international Islet Autoantibody Standardization Program, with an extensive validation exercise performed on paired samples in an external laboratory (Royal Devon and Exeter NHS Foundation Trust, UK) to ensure robust results.

Despite these strengths, the study had some limitations. Participants were recruited only from tertiary hospitals. This, to an extent, introduces selection bias, which affects the generalisability of study findings to the adult Ugandan population with diabetes. However, it is important to note that these facilities serve the general surrounding population and the majority of patients self-refer to the diabetes clinics for management. In addition, total body fat and visceral fat as markers of body adiposity were assessed using bioimpedance analysis, which is a less sensitive approach and has not been widely validated in adult African populations with type 2 diabetes.

### Conclusion

Our study has shown that approximately one-third of patients with adult-onset diabetes were lean (BMI <25 kg/m^2^) and that, pathophysiologically, features of reduced pancreatic beta cell secretory capacity predominate with little contribution from increased total body and visceral adiposity and insulin resistance.

The mechanisms explaining the observed pancreatic secretory dysfunction need to be rigorously investigated, and additional studies are required to develop individualised therapeutic approaches to improve management of lean patients with type 2 diabetes.

## Data Availability

The data are available on request from the authors.

## Abbreviations

GADA: GAD autoantibodies
IA-2A: Autoantibody to the protein tyrosine phosphatase
IGI: Insulinogenic index
WHtR: Waist circumference:height ratio
ZnT8-A: Zinc transporter eight autoantibodies
